# Bayesian network structure for predicting local tumor recurrence in rectal cancer patients treated with neoadjuvant chemoradiation followed by surgery

**DOI:** 10.1016/j.phro.2022.03.002

**Published:** 2022-03-29

**Authors:** Biche Osong, Carlotta Masciocchi, Andrea Damiani, Inigo Bermejo, Elisa Meldolesi, Giuditta Chiloiro, Maaike Berbee, Seok Ho Lee, Andre Dekker, Vincenzo Valentini, Jean-Pierre Gerard, Claus Rödel, Krzysztof Bujko, Cornelis van de Velde, Joakim Folkesson, Aldo Sainato, Robert Glynne-Jones, Samuel Ngan, Morten Brændengen, David Sebag-Montefiore, Johan van Soest

**Affiliations:** a Department of Radiation Oncology (MAASTRO), GROW-School for Oncology and Developmental Biology, Maastricht University Medical Center, Maastricht, The Netherlands; b Fondazione Policlinico Universitario A. Gemelli IRCCS, Roma, Italia; c Universita Cattolica del Sacro Cuore, Roma, Italy; d Department of Radiation Oncology, Gachon University, College of Medicine, Gil Medical Center, Incheon, South Korea; e Department of Radiotherapy, Centre Antoine-Lacassagne, Nice, France; f Department of Radiotherapy, University of Frankfurt, Germany; g Department of Radiotherapy I, M. Skłodowska-Curie National Research Institute of Oncology, Warsaw, Poland; h Department of Surgery, Leiden University Medical Center, The Netherlands; i Department of Surgery, Uppsala University Hospital, Uppsala, Sweden; j Department of Radiotherapy, Pisa University, Italy; k Department of Radiotherapy, Mount Vernon Cancer Centre, Northwood, United Kingdom; l Department of Radiation Oncology, Peter MacCallum Cancer Centre, Melbourne, Victoria, Australia; m Department of Oncology, Oslo University Hospital, Oslo, Norway; n Leeds Institute of Medical Research, University of Leeds, Leeds, United Kingdom

## Abstract

**Background and Purpose:**

Tumor recurrence, a characteristic of malignant tumors, is the biggest concern for rectal cancer survivors. The epidemiology of the disease calls for a pressing need to improve healthcare quality and patient outcomes. Prediction models such as Bayesian networks, which can probabilistically reason under uncertainty, could assist caregivers with patient management. However, some concerns are associated with the standard approaches to developing these structures in medicine. Therefore, this study aims to compare Bayesian network structures that stem from these two techniques.

**Patients and Methods:**

A retrospective analysis was performed on 6754 locally advanced rectal cancer (LARC) patients enrolled in 14 international clinical trials. Local tumor recurrence at 2, 3, and 5-years was defined as the endpoints of interest. Five rectal cancer treating physicians from three countries elicited the expert structure. The algorithmic structure was inferred from the data with the hill-climbing algorithm. Structural performance was assessed with calibration plots and area under the curve values.

**Results:**

The area under the curve for the expert structure on the training and validation data was above 0.9 and 0.8, respectively, for all the time points. However, the algorithmic structure had superior predictive performance over the expert structure for all time points of interest.

**Conclusion:**

We have developed and internally validated a Bayesian networks structure from experts’ opinions, which can predict the risk of a LARC patient developing a tumor recurrence at 2, 3, and 5 years. Our result shows that the algorithmic-based structures are more performant and less interpretable than expert-based structures.

## Highlights

• Algorithmic-based BN structures are more performant than expert structure.
• Algorithmically derived BN structures are comparable to a black-box model.
• The alignment of BN structures with clinical processes increases interpretability.

## 1 Introduction

The introduction of total mesorectal excision (TME) surgery and the use of neoadjuvant chemoradiation (nCRT) have reduced mortality and recurrence rate for rectal cancer patients, with an incidence of locoregional relapses after treatments of 4–8% [1], [2], [3]. Despite the low incidence, tumor recurrence remains the predominant concern for most rectal cancer survivors considering the relatively poor quality of life involved [4], [5]. Besides the treatment procedure, several other factors such as tumor site, size, ethnicity, genetics, etc. could influence the chance of tumor recurrence [5], [6], [7] , and processing all these pieces of information to estimate the likelihood of a patient developing a tumor recurrence after treatment can be overwhelming, even for experts [8], [9]. Predictive models such as Bayesian networks, which consider causal relationships between features, can, on the other hand, learn efficiently from large and heterogeneous volumes of available information and make inferences about future patients.

Bayesian networks are suitable for clinical applications because they can probabilistically reason under uncertainty with an intuitive clinical interpretation of the results [10], [11], [12]. Generally, they can be specified by an expert in the domain of interest or inferred from available data via a learning algorithm [11], [13]. However, these methods may be challenging in healthcare. An algorithm-based structure can include spurious relationships that are not plausible or have no clinical meaning (e.g., causally linking gender to age) due to correlations in the data and the impossibility to determine the direction of causality from data [14]. On the other hand, a structure specified by an expert might be biased by the expert’s prior knowledge and subjective domain experience.

One possible solution to this problem is to survey multiple experts’ opinions. This study hypothesizes that eliciting multiple experts’ opinions will give a reliable Bayesian network structure to predict local tumor recurrences at several time-points (2, 3, and 5 years) in rectal cancer patients whose predictions closely approximate the ground truth. To test this hypothesis, we implemented a solution to examine experts’ opinions on the causal relationships to predict tumor recurrences in locally advanced rectal cancer (LARC) patients.

## 2 Materials and methods

**Fig. 1:**
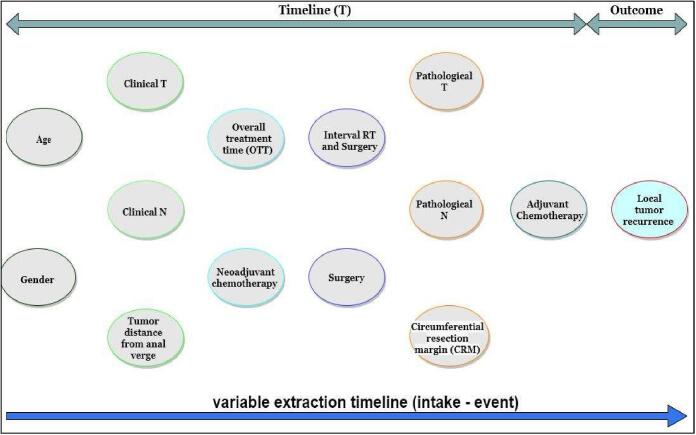
Variables under investigation on extraction timeline.

A retrospective cohort of 6,754 diagnosed LARC patients treated with neoadjuvant chemoradiation followed by surgery from 1993 to 2014 from 14 international trial cohorts was analyzed for this study (Table S1 supplemental material). All the trials have different treatment protocols, patient characteristics, and accrual start dates. Only non-metastatic rectal cancer patients treated with conventional preoperative radiotherapy were considered for this study. Patients with a surgical procedure different from anterior-resection or abdominoperineal resection, treated with adjuvant radiotherapy or incomplete radiotherapy treatment, were excluded due to their relatively low representation. Fig. 1 shows the variables under investigation in this study based on a timeline (**T**) of clinical practice availability. Local tumor recurrence at 2, 3, and 5 years was considered the endpoints of interest defined as detecting a tumor on the same sites it previously started after therapy.

### 2.1 Statistics

Data from the 14 trial cohorts were merged and split into training and validation sets by performing a random 80–20% split (stratified per cohort). The SMOTE algorithm [15] was used to address the class imbalance per response time point, and continuous variables were categorized based on literature and experts’ suggestions. The circumferential resection margin (CRM) was dichotomized into positive if the tumor is ≤1 mm from the circumferential margin and negative if >1 mm (Table S2 supplemental material). Missing values were considered as a category (Unknown) for all variables. However, patients with missing information on their local recurrence status were excluded from further analyses. All analyses were conducted in R version 3.6.1 [16] using the bnlearn package [17], and GeNIe (Graphical Network Interface) application [18] was used for structural visualization. Model performance was assessed by generating calibration plots, model accuracy, and calculating the area under the curve (AUC) on training and validation sets for all time points of interest.

The median age of the 5404 patients in the training and 1350 in the validation cohorts was 61 (22–90) and 61 (25–84) years. Patients’ characteristics and treatment modalities are shown in Table 1.

**Table 1:** General patient characteristics on the training and validation datasets.

Variable	Levels	Training	Validation	p-value
Age (years)	Mean (sd)	61.4 (9.6)	61.4 (10)	0.82
	*Missing*	92 (1.7%)	17 (1.3%)	

Gender	Male	3760 (69.6%)	929 (68.8%)	0.52
	Female	1630 (30.2%)	420 (31.1%)	
	*Missing*	14 (0.2 %)	01 (0.1%)	

Clinical T	cT 1	93 (1.7%)	30 (2.2%)	0.13
	cT 2	387 (7.2%)	117 (8.7%)	
	cT 3	4002 (74.1%)	987 (73.1%)	
	cT 4	370 (6.8%)	84 (6.2%)	
	*Missing*	552 (10.2%)	132 (9.8%)	

Clinical N	cN 0	1547 (28.6%)	367 (27.2%)	0.57
	cN 1	1707 (31.6%)	438 (32.4%)	
	cN 2	303 (5.6%)	78 (5.8%)	
	*Missing*	1847 (34.2%)	647 (34.6%)	

Radiotherapy dose (Gy)	Mean (sd)	47.7 (3.6)	47.7 (3.5)	0.98
	*Missing*	1378 (22.5%)	347 (22.9%)	

Surgery procedure	APR	1629 (30.1%)	426 (31.6%)	*0.42*
	ARbased	3489 (64.6%)	851 (63.0%)	
	No surgery	107 (2.0%)	22 (1.6%)	
	*Missing*	179 (3.3%)	51 (3.8%)	

Circumferential resection margin	Negative	543 (10.1%)	140 (10.3%)	0.91
	Positive	435 (8.0%)	114 (8.4%)	
	*Missing*	4426 (81.9%)	1096 (81.2%)	

Overall treatment time (days)	Mean (sd)	37 (6.6)	37.4 (9.3)	0.16
	*Missing*	1598 (26.1%)	396 (25.8%)	

Neoadjuvant chemo	5FU + OXI	1128 (20.9%)	266 (19.7%)	0.60
	5FUbased	2806 (51.9%)	709 (52.5%)	
	No Chemo	1245 (23.0%)	321 (23.8%)	
	*Missing*	225 (4.2%)	54 (4.0%)	

Tumor distance^d^ (cm)	Mean (sd)	06 (3.1)	06 (3.1)	0.84
	*Missing*	1023 (16.7%)	260 (17.0%)	

Interval between radiotherapy and Surgery (weeks)	Mean (sd)	0.9 (0.4)	0.9 (0.3)	0.77
	*Missing*	2251 (36.7%)	554 (36.2%)	

Adjuvant Chemo	5FU + OXI	651 (12.0%)	152 (11.3%)	0.70
	5FUbased	2497 (46.2%)	621 (46.0%)	
	No Chemo	2024 (37.5%)	515 (38.1%)	
	*Missing*	232 (4.3%)	62 (4.6%)	

Pathological N	ypN 0	3436 (63.6%)	852 (63.1%)	0.95
	ypN 1	1225 (22.7%)	311 (23.0%)	
	ypN 2	312 (5.7%)	77 (5.7%)	
	*Missing*	431 (8.0%)	110 (8.2%)	

Pathological T	ypT 0	625 (11.5%)	148 (11.0%)	0.05
	ypT 1	307 (5.7%)	95 (7.0%)	
	ypT 2	1453 (26.9%)	387 (28.7%)	
	ypT 3	2413 (44.7 %)	557 (41.3%)	
	ypT 4	175 (3.2 %)	53 (3.9%)	
	*Missing*	431 (8.0%)	110 (8.1%)	

2 years local recurrence	True	385 (7.1%)	90 (6.7%)	0.49
	False	4168 (77.1 %)	1060 (78.5%)	
	*Missing*	851 (15.8%)	200 (14.8%)	

3 years local recurrence	True	487 (9.0%)	118 (8.8%)	0.61
	False	3445 (63.8%)	882 (65.3%)	
	*Missing*	1472 (27.2%)	350 (25.9%)	

5 years local recurrence	True	599 (11.1%)	153 (11.3%)	0.66
	False	2036 (37.7%)	497 (36.8%)	
	*Missing*	2769 (51.2%)	700 (51.9%)	

**sd =** standard deviation, **d =** Distance to anal verge (cm), **Chemo =** Chemotherapy.

**APR =** Abdominoperineal resection, **ARbased =** Anterior resection, OXl = oxaliplatin, **5FU =** 5-Fluorouracil.

### 2.2 Structure learning

The domain knowledge from multiple experts’ in three international radiotherapy institutions (Gemelli, Maastro, and Gil Medical Center) was employed to develop and validate the Bayesian network structure. Two experts from Gemelli independently defined the causal relationship between the variables. These experts were requested to draw arrows between variables to indicate causal relationships without setting the relationships’ importance. Relationships were restricted to only variables in the same time-point **t** or the next **t** + n. Arrows drawn from a given variable at time-point **t** to another variable in a preceding time-point **t** - n were considered invalid. For example, arrows from Clinical T to Clinical N stage or from **Age** to **Clinical T stage** are acceptable. However, arrows from **Clinical T stage** to **Age** are rejected.

Two other experts from Maastro separately reviewed the subset of connections common to both experts from Gemelli. The Dutch experts were tasked to validate the relationship by agreeing or disagreeing with each of the connections between the variables made by Gemelli experts. Only connections where both experts agreed were considered for further evaluation. As a final validation, an expert from Gil Medical Center reviewed the subset of connections common to both experts from Maastro. Only the connections validated by the expert were used to construct the final structure. The expert-developed structure was checked for cycles, which are not allowed in Bayesian network structures.

### 2.3 Structure comparison

In order to compare the performance of the developed Bayesian network structure in predicting local recurrence in rectal cancer patients, a structure was also inferred solely from the data with the hill climbing (HC) algorithm [19] for each time point of interest using the same training and validation data as the expert structure. The structures were first compared structurally and then numerically using the AUC, sensitivity, and specificity values. Calibration plots that measure how similar the distribution and behavior of the predicted probabilities are to that observed in data were produced to further evaluate the performance of the structure. The HC algorithm, which looks for the best structure over the search space by adding, removing, and reversing arcs (arrows) in the DAG one at a time, was preferred because it is computationally efficient, and a random restart search was implemented to prevent the structure from getting stuck on a bad local optimum [20]. The Bayesian Information Criterion (BIC), a statistical goodness-of-fit measure that penalizes structural complexity, was used for the structure-learning process [19].

## 3 Results

**Fig. 2:**
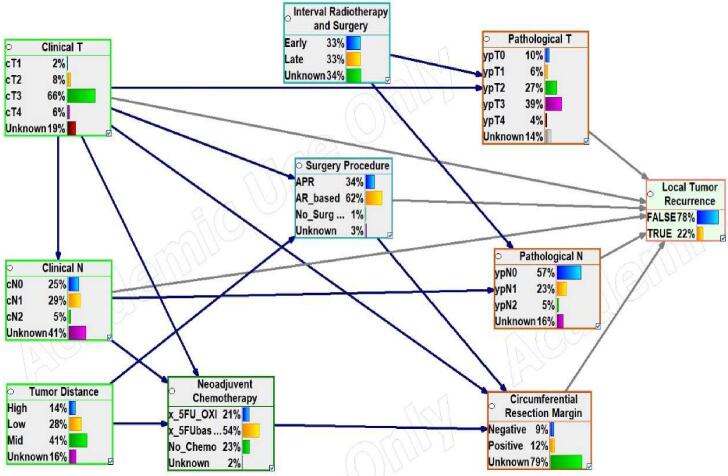
Bayesian network structure based on expert knowledge. The boxes represent the variables (Node); the colors represent the variables’ time points (**t**) of availability in the clinical process, as shown in Fig. 1. The arrows indicate cause-effect relationships. The gray arrows indicate a direct causal effect on the outcome of interest.

Fig. 2 shows the resulting Bayesian network structure based on expert knowledge. This network achieved AUCs above 0.9 and 0.8 for predicting the risk of local recurrence on the training and validation data, respectively, for all time points of interest. Table 2 shows the accuracies, AUCs, and confidence intervals of the structure’s performance on the training and validation data for the three-time points of interest.

**Table 2:** The performance of the expert structure based on the accuracy and AUC values at different time points on the training and validation data.

Time	**Training**			**Validation**		
	Accuracy	AUC	95% CI	Accuracy	AUC	95% CI
2 years	0.84	0.92	0.91–0.92	0.75	0.87	0.85–0.88
3 years	0.83	0.91	0.91–0.92	0.73	0.85	0.84–0.87
5 years	0.83	0.91	0.915–0.92	0.71	0.80	0.78–0.81

**CI** = confidence interval.

### 3.1 Structure comparison

The Bayesian network structures resulting from the HC algorithm mentioned above used all 14 available variables (Fig. S1 supplemental material) with 32, 33, and 30 arcs for 2, 3, and 5 years tumor recurrence, respectively. On the other hand, the experts’ structure had 19 arcs and 10 variables, excluding age, gender, adjuvant chemotherapy, and overall treatment time. The outcome had a direct patent-to-child connection with all 13 nodes for the structure at 5-years, and the outcome for the 2-year structure had 11 children, excluding the arc with adjuvant chemotherapy and CRM, while that of the 3-year structure had 10, excluding adjuvant chemotherapy, pathological T, and N which was quite the opposite for the expert structure with just six parents. The only similarity between the algorithmic and expert structures was the arc CRM to the outcome for the 2-years structure and the arcs pathological T and N to the outcome for the 3-years structure.

Based on the relationship of the variables with the outcome among the algorithmic structures, the arcs pathological T and N to the outcome for the 3-years structure were reversed in the 2, and 5-year structures, and the arc CRM to the outcome in the 2-year structure was reversed in the 3, and 5-year structures while the arc adjuvant chemotherapy to the outcome in the 2-year structure was reversed in the 5-year structure and absent in the 3-year structure. Age was only connected to the outcome alone for 3 and 5 years structures.

The numerical comparison of the expert and algorithmic structures based on their performance in terms of the AUC ( Fig. S2 supplemental material), sensitivity, and specificity values for each time points in the training and validation data is shown in Table 3. The algorithmic structures had a slightly better performance than the expert structure for all three matrices of interest, especially in the validation data. However, the difference in the AUC values between the structures was not statistically significant (p-value >0.05) for all time points.

**Table 3:** The AUC, sensitivity, and specificity values of the expert and algorithmic structures on the training and validation data at different time points.

**Training**				
**Timepoint**	**BN Structure**	**AUC value**	**Sensitivity**	**Specificity**
2 years	Experts	0.92	0.93	0.75
	Algorithm	0.93	0.92	0.78
3 years	Experts	0.91	0.91	0.75
	Algorithm	0.93	0.91	0.79
5 years	Experts	0.91	0.91	0.75
	Algorithm	0.93	0.91	0.80

**Validation**				
2 years	Experts	0.87	0.85	0.65
	Algorithm	0.89	0.88	0.74
3 years	Experts	0.85	0.84	0.62
	Algorithm	0.91	0.88	0.75
5 years	Experts	0.80	0.73	0.70
	Algorithm	0.88	0.96	0.71

**Fig. 3:**
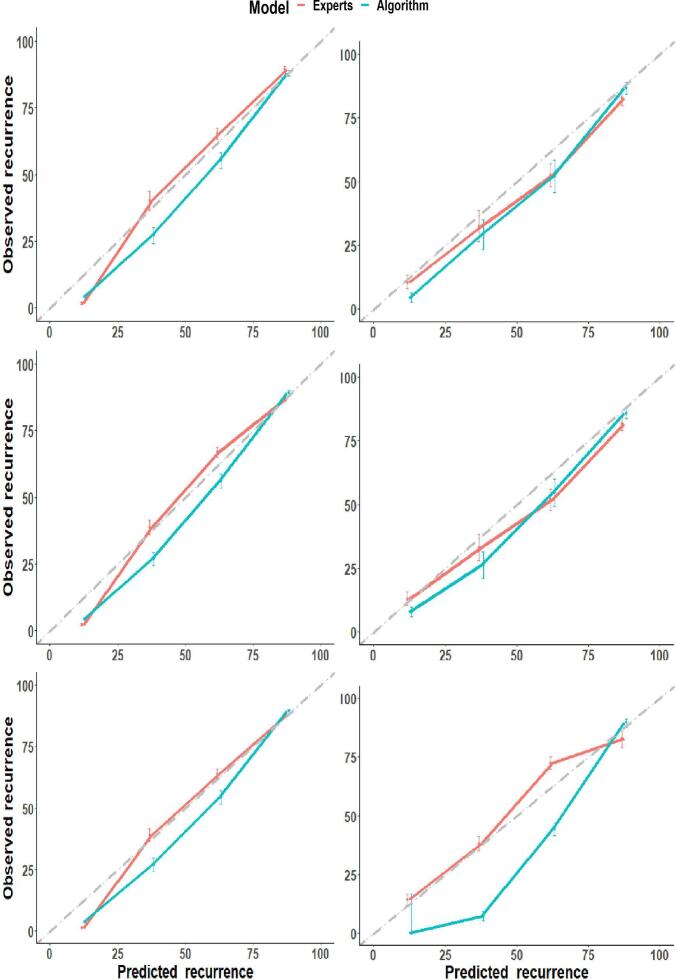
Calibration plots of the models on the training (left) and validation (right) data for 2-year (top) to 5-year (bottom) local recurrence. The gray dashed line represents ideal calibration, while solid lines represent each model’s calibration. Vertical bars indicate a 95% confidence interval, and dots indicate bias-corrected estimates.

The calibration plots in Fig. 3 show a good match between the predicted probabilities and the observed frequencies in the training and validation cohort for both expert and algorithmic structures. Generally, the expert structure seems to be better calibrated than the algorithmic structures since most points are closer to the dotted diagonal gray line, representing an ideal or perfect structure, especially recurrence at 5-years.

## 4 Discussion

In the current study, we have developed and internally validated a Bayesian network structure based on five experts’ opinions from three different radiation therapy treatment institutions to predict tumor recurrence for locally advanced rectal cancer patients. The structure was developed to capture the biological process that leads to a tumor recurrence by connecting the variables in the structure based on a timeline of their clinical availability. The developed structure used nine clinical features well-known to clinicians as pivotal factors for predicting local tumor recurrence. The structure was well-calibrated with predictive performance based on the AUC values above 0.9 and 0.8 in training and validation data, respectively, for all time points of interest. Structures inferred from a learning algorithm used four variables more than the expert structure, including age, gender, adjuvant chemotherapy, and overall treatment time, with slightly better performance in terms of AUC values. However, the expert structure was clinically more plausible than the algorithmic structures and aligned with the clinical process.

The choice of model to predict local tumor recurrence in rectal cancer patients in this study was influenced by two main reasons. Firstly, Bayesian networks better represent complex systems such as the clinical processes leading to tumor recurrence since they have more liberty to define interactions between variables [10], [21], unlike the generally used regression method [22], [23], [24]. Secondly, their ability to make inferences on any variable(s) in the network makes them very valuable for decision support as they can serve as a diagnostic and prognostic tool. Thus far, this study is the first to assess the predictive value of Bayesian networks for tumor recurrence in rectal cancer patients which makes a direct comparison and interpretation with other studies arduous because of the difference in analytical approach and study design.

Nonetheless, Valentini et al. [22] previously developed a model to predict tumor recurrence in rectal cancer patients as a decision support tool. The model included age as a predictive factor for local recurrence, a variable missing in our expert structure. One apparent reason for this difference is the variable selection procedure, given that age is also present in the algorithmic structures. Algorithm-based variable selection methods exploit spurious correlations within the data, which might unnecessarily increase the complexity of the model and cause overfitting. Also, Farhat et al. [25] did not find age or gender a predictive factor for tumor recurrence in their 16-year respective study, which is in support of the expert structure. Although the controversy of included variables depends largely on patient heterogeneity between the studies, variable inclusion in a prediction model must not always be strictly algorithm-based, even if it improves its performance. Instead, it should contain some level of clinical understanding, context, or rationale, which domain experts are more suited to provide because a correlation between variables does not necessarily imply causality.

The algorithmic structures use four variables more than the expert structure, which explains its slightly better predictive performance. However, the additional variables increase the complexity of the structure since more connections between the variables are formed but with minimal predictive benefits (Table 3). Although the expert structure did not perform better than the algorithmic structures, we believe the expert structure might be more suitable for clinical use over the algorithmic structure. Firstly, the algorithmic structure uses parent-to-child connections not aligned with the clinical process (e.g., the pathological nodal stage has a causal link to age at start radiotherapy) to make decisions. These unconformable arcs imply that algorithmically generated structures are comparable to black-box models even with better performance on both data sets since the decision process lacks clinical explanation. Regarding model calibration, the expert structure seems to be better calibrated for all time points than the algorithmic structure with higher AUCs.

Tumor recurrence is a very challenging endpoint not only in terms of quality of life for cancer survivors [4], [5] but also the difficulty in accurately predicting the endpoint [26]. Patient variability can explain this difficulty since a treatment regime that leads to recurrence-free for one patient might not give another patient the same outcome. Therefore, collaboration with domain experts is pivotal to have a more personalized prediction of tumor recurrence since they better understand tumor biology. The performance of the proposed expert structure is well above the chance level with clinically valid relationships. Therefore, it might be valuable in routine clinical settings as a decision aid to support personalized treatment decision-making. Also, it could guide clinicians to opt for a more aggressive adjuvant therapy to prevent the chance of a tumor recurrence for patients who have undergone surgery but with a high predicted probability of a tumor recurrence in the structure. However, the structure is trained on retrospective clinical trial data and warrants an external validation on routine clinical data to ascertain its clinical usefulness. In addition, the circumferential resection margin, a variable proven to influence local tumor recurrence [27], [28], had a large proportion of missing information and will be worthwhile to retrain its conditional probabilities on a more complete dataset.

Despite the predictive performance of the Bayesian network structures in this study, there is still room for improvement. The international and multi-trial nature of our study may be seen as a limitation, given that it combines the contribution of experts from three cancer institutions and data from multiple clinical trials with different treatment protocols. However, this limitation could also be considered a strength, as it may make our findings more robust and generalizable. The multi-trial combination is particularly relevant for this study since it enables the structure to be trained on a large sample size, which reflects the models’ superior performance over other studies with relatively smaller sample sizes [22], [23], [24] given that model performance is proportional to training sample size. Also, this large sample size helped improve the number of events given the disease’s low event rate. Despite combining data from 14 different European trials, the number of local recurrence events was relatively low. Our study design also prevents updating the number of connections between variables since each expert is contacted only once for input. Also, some of the variables were categorized, leading to a loss of information. Lastly, blood tumor markers such as carcinoembryonic antigen (CEA), a proven predictive factor for local tumor recurrence in rectal cancer [25], were not included in the structure because of the study’s retrospective nature.

In summary, we have developed and validated a Bayesian network structure from 14 trial cohorts’ data by analyzing a total number of 6754 rectal cancer patients for predicting the risk of local recurrence in locally advanced rectal cancer patients at 2, 3, and 5 years. The causal relationships between the variables in the developed Bayesian network structure were proposed and validated by domain experts with years of experience from different international radiotherapy centers, where treatment protocols may differ. Our result showed that although structures from both methods performed above chance level, the algorithmic-based structures had higher discriminating power than the expert structure. However, they contained clinically incomprehensible arcs, making the expert structure more credible even with relatively lower predictive performance. Future research will combine these two Bayesian network structure learning approaches to produce clinically plausible structures with optimal predictive performance.

## Declaration of Competing Interest

The authors declare that they have no known competing financial interests or personal relationships that could have appeared to influence the work reported in this paper.

## References

[1] Sebag-Montefiore D., Stephens R.J., Steele R., Monson J., Grieve R., Khanna S. (2009). Preoperative radiotherapy versus selective postoperative chemoradiotherapy in patients with rectal cancer (MRC CR07 and NCIC-CTG C016): a multicentre, randomised trial. The Lancet.

[2] Kapiteijn E., Marijnen C.A., Nagtegaal I.D., Putter H., Steup W.H., Wiggers T. (2001). Preoperative radiotherapy combined with total mesorectal excision for resectable rectal cancer. N Engl J Med.

[3] Ikoma N., You Y.N., Bednarski B.K., Rodriguez-Bigas M.A., Eng C., Das P. (2017). Impact of recurrence and salvage surgery on survival after multidisciplinary treatment of rectal cancer. J Clin Oncol.

[4] Baker F., Denniston M., Smith T., West M.M. (2005). Adult cancer survivors: how are they faring?. Cancer.

[5] Denlinger C.S., Barsevick A.M. (2009). The challenges of colorectal cancer survivorship. J Natl Compr Canc Netw.

[6] Mannell A. (2017). An overview of risk factors for recurrent breast cancer. S Afr J Surg.

[7] Zare-Bandamiri M., Fararouei M., Zohourinia S., Daneshi N., Dianatinasab M. (2017). Risk factors predicting colorectal cancer recurrence following initial treatment: a 5-year cohort study”. Asian Pac J Cancer Prev.

[8] Abernethy A.P., Etheredge L.M., Ganz P.A., Wallace P., German R.R., Neti C. (2010). Rapid-learning system for cancer care. J Clin Oncol.

[9] Oberije C., Nalbantov G., Dekker A., Boersma L., Borger J., Reymen B. (2014). A prospective study comparing the predictions of doctors versus models for treatment outcome of lung cancer patients: a step toward individualized care and shared decision making. Radiother Oncol.

[10] Korb K.B., Nicholson A.E. (2010).

[11] Jayasurya K., Fung G., Yu S., Dehing-Oberije C., De Ruysscher D., Hope A. (2010). Comparison of Bayesian network and support vector machine models for two-year survival prediction in lung cancer patients treated with radiotherapy. Med Phys.

[12] Sesen M.B., Nicholson A.E., Banares-Alcantara R., Kadir T., Brady M. (2013). Bayesian networks for clinical decision support in lung cancer care. PLoS ONE.

[13] Jochems A, Deist TM, El Naqa I, Kessler M, Mayo C, Reeves J, et al. Developing and validating a survival prediction model for NSCLC patients through distributed learning across 3 countries. *Int J Radiat Oncol Biol Phys* 2017:99;344–352. DOI: 10.1016/j.ijrobp.2017.04.021. 10.1016/j.ijrobp.2017.04.021 PMC5575360 28871984

[14] Pearl J. Introduction to probabilities, graphs, and causal models. In: *Causality: models, reasoning and inference* (2000), pp. 1–40. DOI: https://doi.org/10.1017/ CBO9780511803161.003.

[15] Torgo L. *Data mining with R: learning with case studies*. Chapman and Hall/CRC, 2011. ISBN: 9780367573980.

[16] R Core Team. *R: A language and environment for statistical computing*. R Foundation for Statistical Computing. Vienna, Austria, 2014. URL: http://www.R-project.org/.

[17] Scutari M. (2010). Learning Bayesian Networks with the bnlearn R Package. J Stat Softw.

[18] Druzdzel MJ. SMILE: Structural Modeling, Inference, and Learning Engine and GeNIe: a development environment for graphical decision-theoretic models. *Aaai/Iaai*. 1999, pp. 902–903. ISBN: 0-262-51106-1.

[19] Neapolitan R.E. (2004).

[20] Gamez J.A., Mateo J.L., Puerta J.M. (2007). *European Conference on Symbolic and Quantitative Approaches to Reasoning and Uncertainty*. Springer.

[21] Lucas P. *Bayesian networks in medicine: a model-based approach to medical decision making*. na, 2001.

[22] Valentini V., Van Stiphout R.G., Lammering G., Gambacorta M.A., Barba M.C., Bebenek M. (2011). Nomograms for predicting local recurrence, distant metastases, and overall survival for patients with locally advanced rectal cancer on the basis of European randomized clinical trials. J Clin Oncol.

[23] Van Gijn W., Van Stiphout R., Van De Velde C., Valentini V., Lammering G., Gambacorta M.A. (2015). Nomograms to predict survival and the risk for developing local or distant recurrence in patients with rectal cancer treated with optional short-term radiotherapy. Ann Oncol.

[24] Hida K., Okamura R., Park S.Y., Nishigori T., Takahashi R., Kawada K. (2017). A new prediction model for local recurrence after curative rectal cancer surgery: development and validation as an Asian collaborative study. Dis Colon Rectum.

[25] Farhat W., Azzaza M., Mizouni A., Ammar H., Lagha S., Kahloul M. (2019). Factors predicting recurrence after curative resection for rectal cancer: a 16-year study. World J Surg Oncol.

[26] Peng J., Ding Y., Tu S., Shi D., Sun L., Li X. (2014). Prognostic nomograms for predicting survival and distant metastases in locally advanced rectal cancers. PLoS ONE.

[27] Warrier S.K., Kong J.C., Guerra G.R., Chittleborough T.J., Naik A., Ramsay R.G. (2018). Risk factors associated with circumferential resection margin positivity in rectal cancer: a binational registry study. Dis Colon Rectum.

[28] Liu Q., Luo D., Cai S., Li Q., Li X. (2018). Circumferential resection margin as a prognostic factor after rectal cancer surgery: A large population-based retrospective study. Cancer Med.

