# Supplementary material for: Bayesian network structure for predicting local tumor recurrence in rectal cancer patients treated with neoadjuvant chemoradiation followed by surgery
Source: Phys Imaging Radiat Oncol. 2022 Mar 29;22:1–7. doi: 10.1016/j.phro.2022.03.002 (PMC8968052; doi:10.1016/j.phro.2022.03.002)
Supplement: Supplementary data 1 [file mmc1.docx]

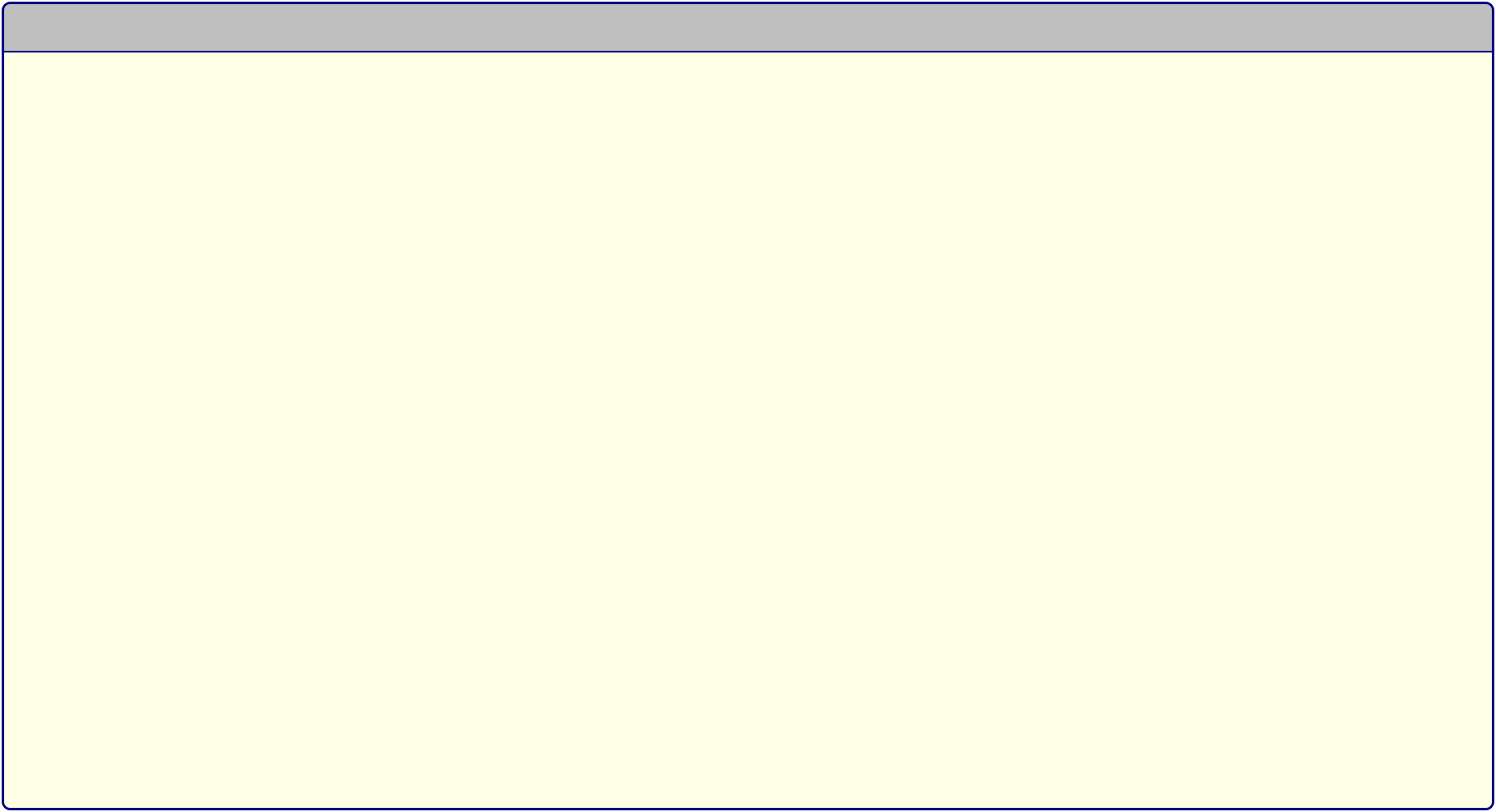


**Supplementary-Table S1.** The 14 clinical trials, accrual dates, and the total number of patients from each trial included

| **Trial** |  | **Accrual** | **Total** |  | **Trial description** |
| --- | --- | --- | --- | --- | --- |
|  |  |  |  |  |  |
|  |  |  |  |  |  |
| [**EORTC-**](https://www.eortc.org/research_field/clinical-detail/22921/) |  |  |  |  | Four arms phase III clinical trial for T3-T4 resectable rectal cancer  comparing preoperative pelvic irradiation to preoperative irradiation  combined with fluorouracil and Leucovorin with or without postoperative  adjuvant chemotherapy. |
|  |  | **1993 - 2003** | **1006** |  |  |
| [**22921-ROG**](https://www.eortc.org/research_field/clinical-detail/22921/) |  |  |  |  |  |
|  |  |  |  |  |  |
|  |  |  |  |  |  |
|  |  |  |  |  |  |
|  |  |  |  |  |  |
| [**Polish I**](https://bjssjournals.onlinelibrary.wiley.com/doi/full/10.1002/bjs.5506) |  | **1999 - 2002** | **139** |  | Long-term results of a randomized trial comparing preoperative short-course  radiotherapy with preoperative conventionally fractionated chemoradiation  for rectal cancer. |
|  |  |  |  |  |  |
|  |  |  |  |  |  |
|  |  |  |  |  |  |
|  |  |  |  |  |  |
| [**ACCORD**](https://ascopubs.org/doi/full/10.1200/JCO.2012.42.8771) |  | **2005 - 2008** | **594** |  | The ACCORD 12 trial investigated the value of two different preoperative  chemoradiotherapy (CT-RT) regimens in T3-4 Nx M0 resectable rectal  cancer. |
|  |  |  |  |  |  |
|  |  |  |  |  |  |
|  |  |  |  |  |  |
|  |  |  |  |  |  |
| [**INTERACT**](https://www.sciencedirect.com/science/article/pii/S0167814018336181) |  | **2006 - 2013** | **476** |  | Long-term results of a randomized trial on preoperative capecitabine based  radiochemotherapy intensified by concomitant boost or oxaliplatin for cT2  (distal)–cT3 rectal cancer. |
|  |  |  |  |  |  |
|  |  |  |  |  |  |
|  |  |  |  |  |  |
|  |  |  |  |  |  |
| [**MRC CR07**](https://www.ncbi.nlm.nih.gov/pmc/articles/PMC2668947/) |  | **1998 - 2005** | **717** |  | Compared short-course preoperative radiotherapy versus initial surgery with  selective postoperative chemoradiotherapy. |
|  |  |  |  |  |  |
|  |  |  |  |  |  |
|  |  |  |  |  |  |
|  |  |  |  |  |  |
| [**FFCD 2903**](https://ascopubs.org/doi/full/10.1200/JCO.2006.06.7629) |  | **1993 - 2003** | **732** |  | Preoperative Radiotherapy With or Without Concurrent Fluorouracil and  Leucovorin in T3-4 Rectal Cancers. |
|  |  |  |  |  |  |
|  |  |  |  |  |  |
|  |  |  |  |  |  |
|  |  |  |  |  |  |
| [**CAO/ARO/AIO-94**](https://ascopubs.org/doi/full/10.1200/JCO.2011.40.1836) |  | **1995 - 2002** | **503** |  | Compared preoperative chemoradiotherapy with postoperative  chemoradiotherapy for locally advanced rectal cancer. |
|  |  |  |  |  |  |
|  |  |  |  |  |  |
|  |  |  |  |  |  |
|  |  |  |  |  |  |

24


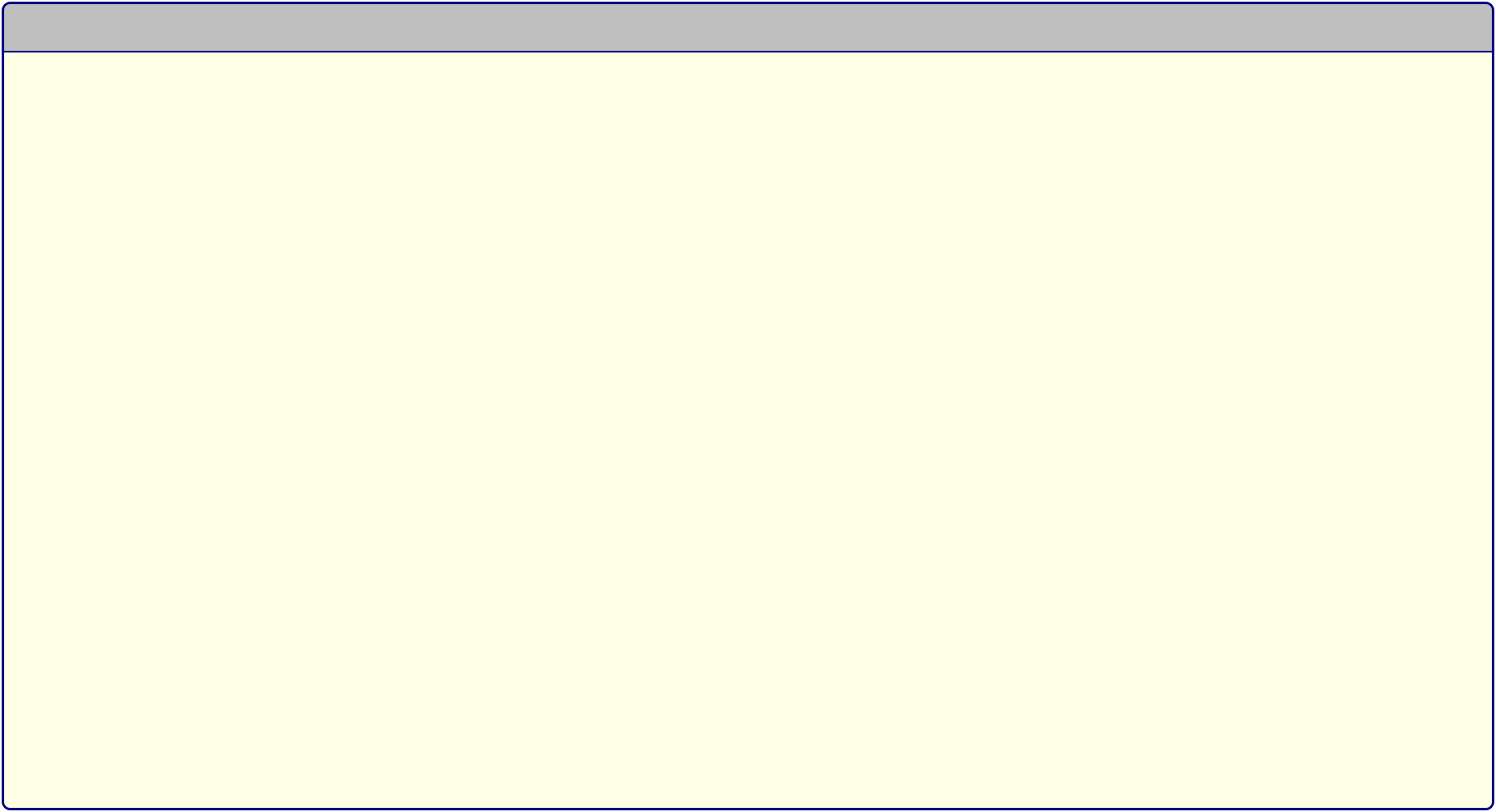


The 14 clinical trials included in this analysis

| **Trial names** |  |  | **Total** |  | **Trial description** |
| --- | --- | --- | --- | --- | --- |
|  |  |  |  |  |  |
|  |  | |  |  |  |
| [**Polish II**](https://www.sciencedirect.com/science/article/pii/S0923753419312967) |  | **2008 - 2014** | **267** |  | Long-course oxaliplatin-based preoperative chemoradiation versus 5X5 Gy  and consolidation chemotherapy for cT4 or fixed cT3 rectal cancer. |
|  |  |  |  |  |  |
|  |  |  |  |  |  |
| [**I-CNR-RT**](https://www.sciencedirect.com/science/article/pii/S0167814014004149) |  | **1992 - 2001** | **605** |  | Evaluate the effect of adjuvant chemotherapy (ACT) in locally advanced  rectal cancer (LARC) after neoadjuvant chemoradiation (NACT-RT). |
|  |  |  |  |  |  |
|  |  |  |  |  |  |
|  |  |  |  |  |  |
|  |  |  |  |  |  |
| [**Nordic**](https://www.sciencedirect.com/science/article/pii/S0167814018302457) |  | **1996 - 2003** | **205** |  | Compared preoperative long-course radiotherapy alone (RT) or with  chemotherapy (CRT) in the most locally advanced/ugly rectal cancers. |
|  |  |  |  |  |  |
|  |  |  |  |  |  |
|  |  |  |  |  |  |
|  |  |  |  |  |  |
| [**Chronicle**](https://www.sciencedirect.com/science/article/pii/S0923753419366967) |  | **2005 - 2008** | **111** |  | Examined the benefit of postoperative adjuvant capecitabine and oxaliplatin  (XELOX) chemotherapy. |
|  |  |  |  |  |  |
|  |  |  |  |  |  |
|  |  |  |  |  |  |
|  |  |  |  |  |  |
| [**CAO/ARO/AIO-04**](https://www.thelancet.com/journals/lanonc/article/PIIS1470-2045(12)70187-0/fulltext) |  | **2008 - 2010** | **1222** |  | Preoperative chemoradiotherapy and postoperative chemotherapy with  fluorouracil and oxaliplatin versus fluorouracil alone in locally advanced  rectal cancer. |
|  |  |  |  |  |  |
|  |  |  |  |  |  |
|  |  |  |  |  |  |
|  |  |  |  |  |  |
|  |  |  |  |  | To document local recurrence in primary rectal cancer when standardized  techniques of surgery, radiotherapy, and pathology are used and to  investigate whether the local recurrence rate after total mesorectal excision  permits the omission of adjuvant short-term preoperative radiotherapy. |
| [**Dutch**](https://onlinelibrary.wiley.com/doi/abs/10.1080/110241599750006613) |  | **1996 - 1999** | **14** |  |  |
|  |  |  |  |  |  |
|  |  |  |  |  |  |
|  |  |  |  |  |  |
|  |  |  |  |  |  |
|  |  |  |  |  |  |
| [**TROG 01-04**](https://ascopubs.org/doi/full/10.1200/JCO.2012.42.9597) |  | **2001 - 2006** | **163** |  | Randomized trial of short-course radiotherapy versus long-course  chemoradiation comparing rates of local recurrence in patients with T3 rectal  cancer. |
|  |  |  |  |  |  |
|  |  |  |  |  |  |
|  |  |  |  |  |  |
|  |  |  |  |  |  |

| **Sup****plementary-Table S2.** Variables discretization. | | | | | | |  |  |  |  |  |
| --- | --- | --- | --- | --- | --- | --- | --- | --- | --- | --- | --- |
| 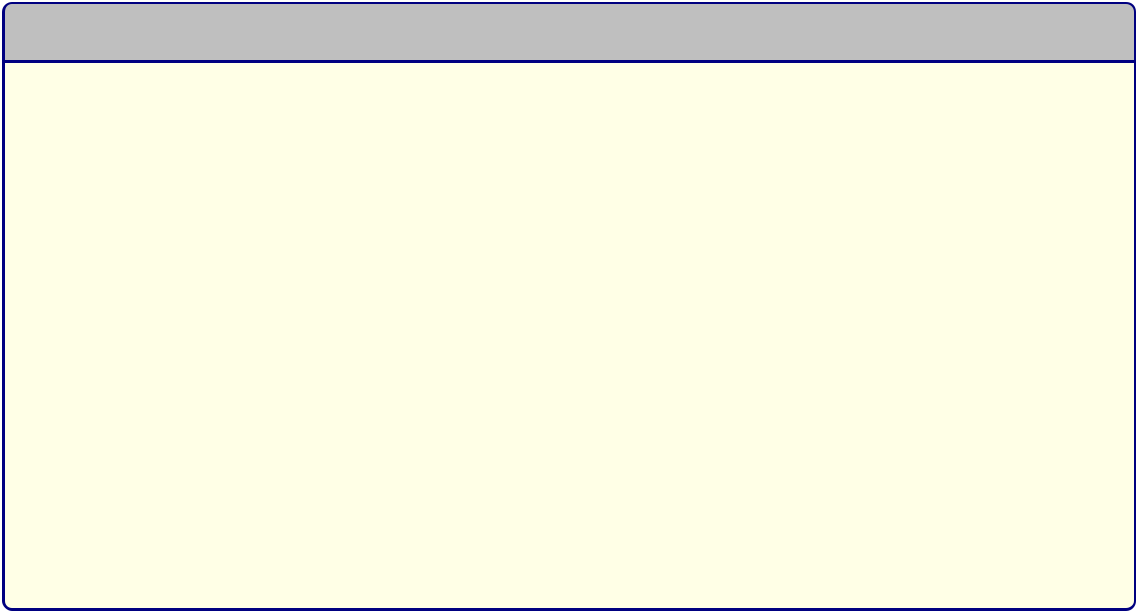**Variable** |  |  |  | **Levels** | | |  |  |  | **Names** |  |
|  |  |  |  |  |  |  |  |  |  |  |  |
|  |  |  |  |  | | |  |  |  |  |  |
|  |  |  |  |  | | |  |  |  |  |  |
|  |  |  |  | Less than 49 years | | |  |  |  | Adults |  |
|  |  |  |  |  | | |  |  |  |  |  |
| Patient Age at Radiotherapy |  |  |  | Between 50 to 59 years | | |  |  |  | Aged |  |
|  |  |  |  | Between 60 to 69 years | | |  |  |  | Old |  |
|  |  |  |  |  |  |  |  |  |  |  |  |
|  |  |  |  | Above 70 years | | |  |  |  | Senior |  |
|  |  |  |  |  | | |  |  |  |  |  |
| The time interval between radiotherapy |  |  |  | Before six weeks | | |  |  |  | Early |  |
| and surgery |  |  |  | After six weeks | | |  |  |  | Late |  |
|  |  |  |  |  | | |  |  |  |  |  |
|  |  |  |  | Less than 5 cm | | |  |  |  | Low |  |
| Tumor distance from the anal verge in cm |  |  |  |  | | |  |  |  |  |  |
|  |  |  |  | Between 5 - 10 cm | | |  |  |  | Mid |  |
|  |  |  |  | Above 10 cm | | |  |  |  | High |  |
|  |  |  |  |  | | |  |  |  |  |  |
| Overall treatment time (OTT) |  |  |  | Less than 37 days | | |  |  |  | Short |  |
|  |  |  |  |  | | |  |  |  |  |  |
|  |  |  |  | More than 37 days | | |  |  |  | Long |  |
|  |  |  |  |  |  |  |  |  |  |  |  |
| Circumferential resection margin (CRM) | |  |  | ≤1mm |  |  |  |  |  | Positive |  |
|  |  |  |  | >1mm | | |  |  |  | Negative |  |
|  |  |  |  |  | | |  |  |  |  |  |

**
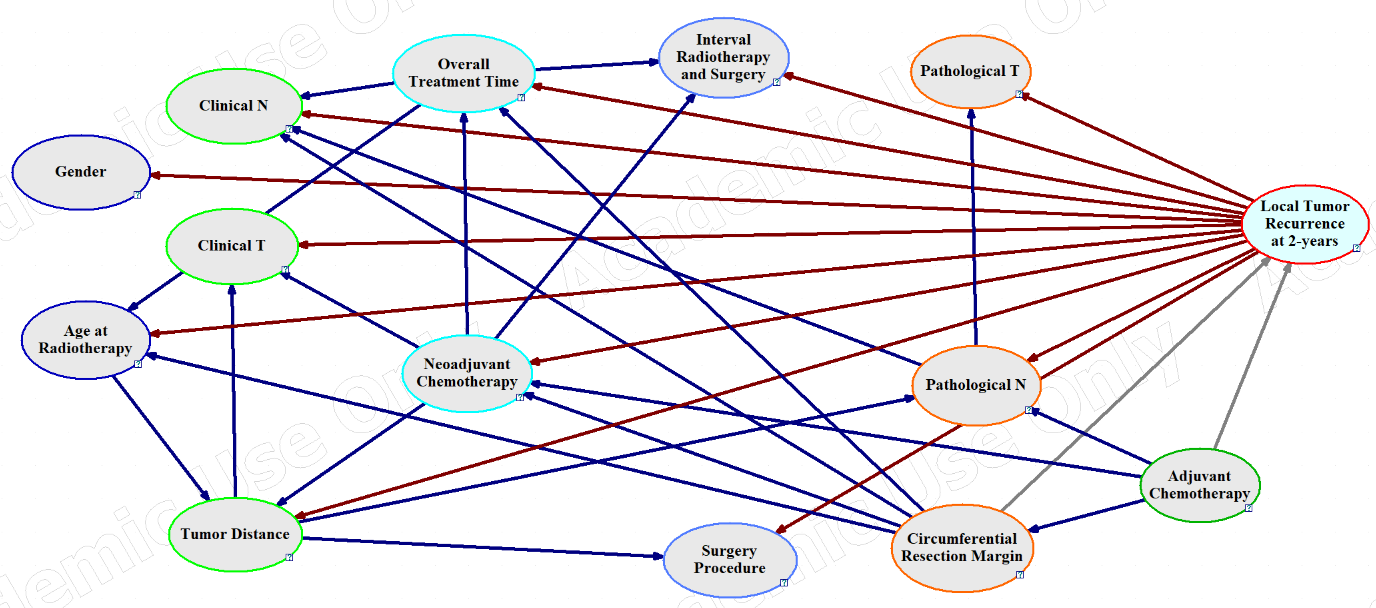

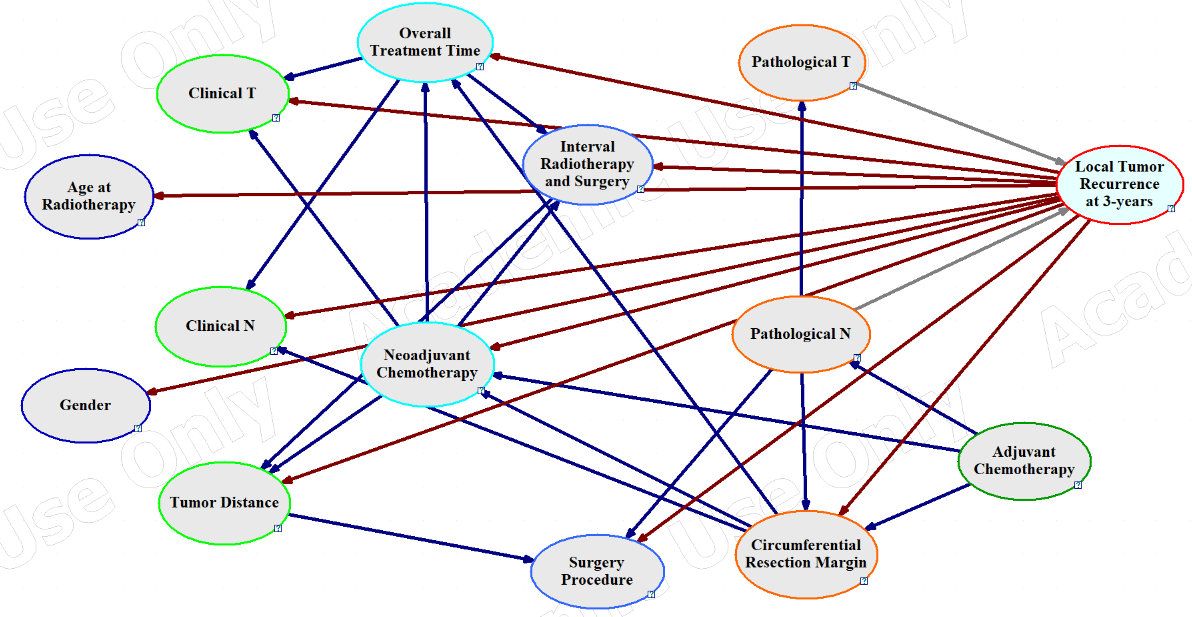
**

**
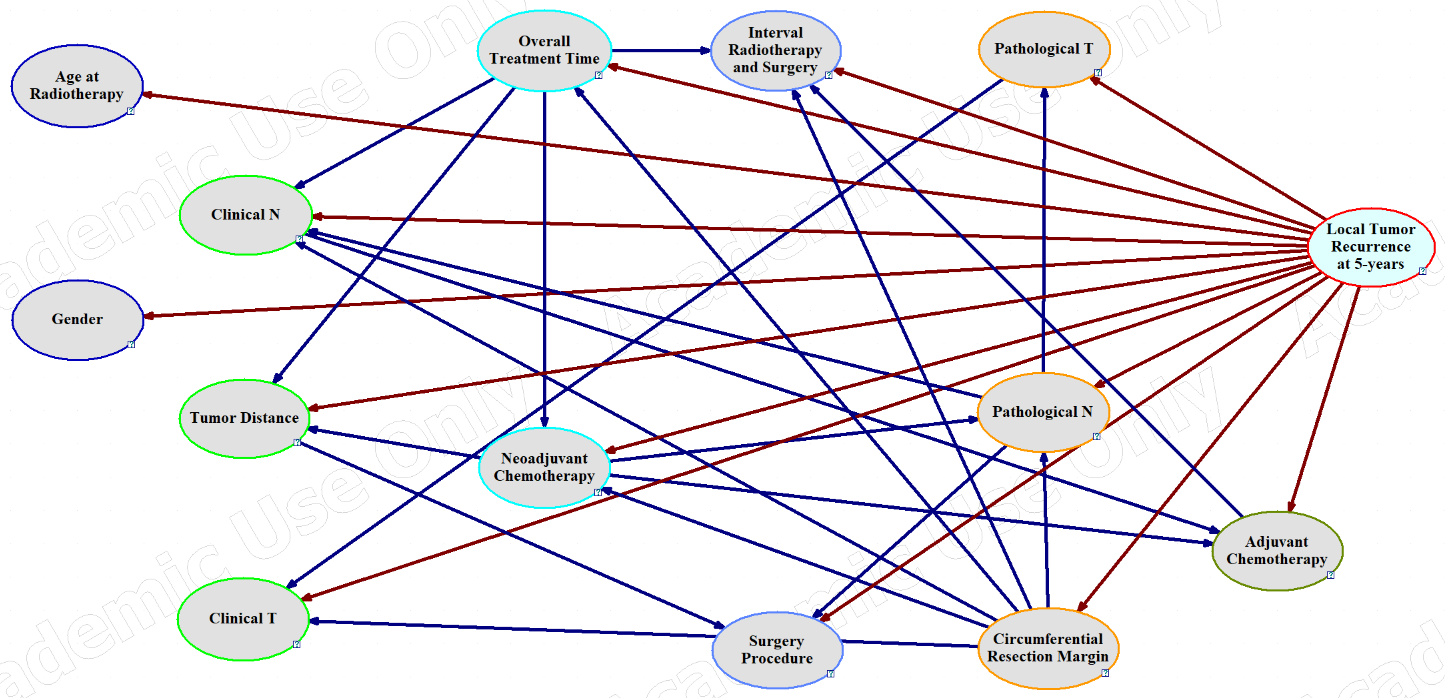
**

**Supplementary-Fig S1.** *Developed Bayesian Network structures based on the hill-climbing algorithm. The circles represent the variables (Node), and* *the arrows indicate the direction of the causal-effect relationships. The brown arrows indicate a direct parental link from the response of interest, and gray arrows indicate a direct* *parental connection to the response.*


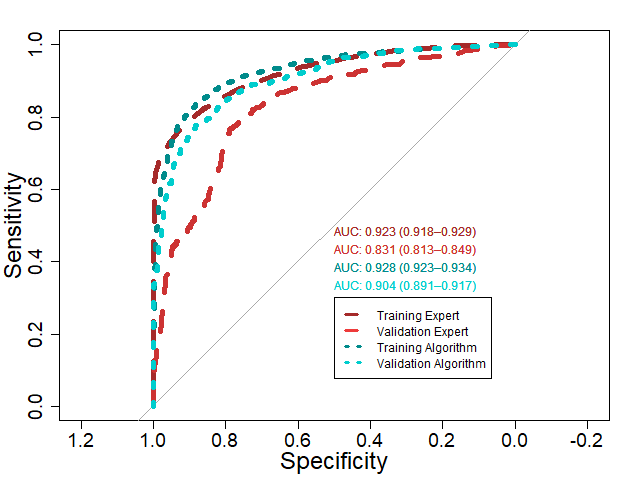

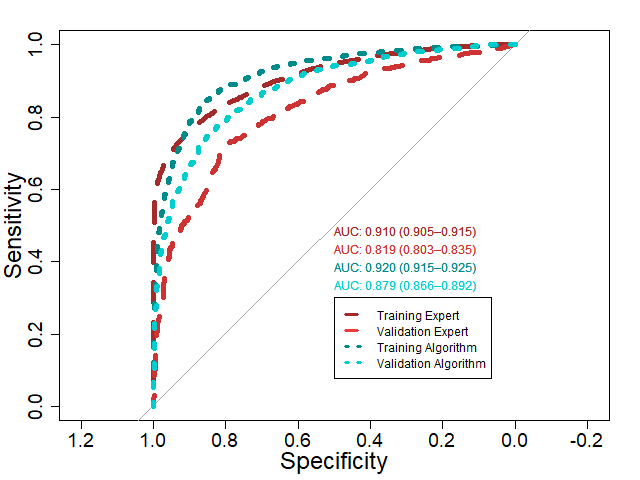

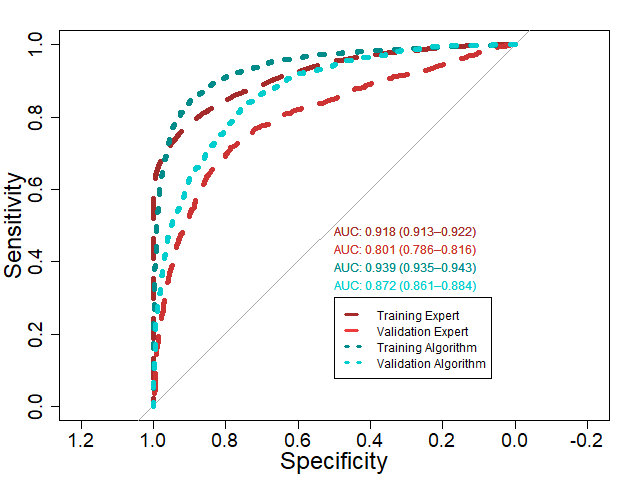


**Supplementary-Fig S2.** *ROC curves for expert (red) and algorithm (cyan) structures on the training and validation data for 2, 3, and 5-years from left to right, respectively.*
